# Online citizen dialogue on biodiversity conservation and citizen participation: A cross-cultural deliberation between Taiwan and Japan

**DOI:** 10.1007/s13280-025-02209-6

**Published:** 2025-07-09

**Authors:** Hidenori Nakamura, Wei-Lin Chen, Fuki Ueno, Satoru Sugita

**Affiliations:** 1 Department of Environmental and Civil Engineering, Toyama Prefectural University, 5180 Kurokawa, Imizu, Toyama 939-0398 Japan; 2 Department of Leisure Industry and Health Promotion, National Taipei University of Nursing and Health Sciences, No. 365, Ming-te Road, Peitou District, Taipei City, 11219 Taiwan, R.O.C.; 3 Department of Social Informatics, Nagoya University, Furo-cho, Chikusa, Nagoya, 464-8601 Japan; 4 Chubu Institute for Advance Studies, Chubu University, 1200 Matsumoto-cho, Kasugai, Aichi 487-8501 Japan

**Keywords:** Deliberative democracy, Open dialogue, Participatory governance, Reflecting, Resilience, Sustainable development goals

## Abstract

This study empirically explored the possibility of nurturing and cultivating reflexivity capacity at the small-group level through cross-cultural online citizen dialogue on biodiversity conservation and citizens’ role using a method of reflecting, connecting Taiwan and Japan. The study found that cross-cultural dialogue improved self-awareness of cultural inheritance as a hindering and promoting factor for sustainability transformation in one’s own cultural community. The dialogue also expanded the perceived boundary of collaboration measured by trust score/ratio. It also brought about the positive change in self-reported attitude toward dialogue with a person holding different views, and of urgency/significance self-assessment of sustainability transformation issues, that is, biodiversity conservation and citizen participation. The institutionalization of cross-cultural citizen dialogue in collective decision-making processes, such as those of the United Nations, and reflexivity capacity building, both at the small-group and population levels through citizen dialogue connecting different cultural groups, are suggested for sustainability transformation.

**Supplementary Information:**

The online version contains supplementary material available at 10.1007/s13280-025-02209-6.

## Introduction

Biodiversity conservation is a key concern for the global community, with several recent advancements having been made in response to continuing global biological degradation. The Kunming–Montreal Global Biodiversity Framework (GBF) was adopted during the fifteenth meeting of the Conference of the Parties to the Convention on Biological Diversity in Montreal in December 2022. At the meeting, the 2050 vision and new global targets to be achieved by 2030, including the effective conservation and management of at least 30% of the world’s land, coastal areas, and oceans, were developed (Convention on Biological Diversity 2022). The 30 × 30 target is expected to be achieved through ecologically representative, well-connected, and equitably governed systems of protected areas and other effective area-based conservation measures. This conservation effort requires citizen participation in ecosystem governance, as stipulated by Target 22 of GBF’s Global Targets for 2030: “Ensure the full, equitable, inclusive, effective and gender-responsive representation and participation in decision-making.” The sixteenth meeting of the Conference of the Parties (COP16) to the Convention on Biological Diversity, held in Columbia in October–November 2024—the first meeting after GBF—achieved some success with outstanding issues of monitoring and financing of GBF, which calls for a stronger support of the people to strive further (United Nations 2024).

Biodiversity conservation in terrestrial and marine ecosystems is envisioned in the United Nations Sustainable Development Goals (SDGs) 15 and 16 (United Nations 2015). To ensure a fair and just transition process of sustainable development, SDG Target 16.7 seeks to ensure “responsive, inclusive, participatory and representative decision-making at all levels.” In the business and finance sectors, biodiversity-related risk management and disclosure institutions were developed alongside carbon disclosure institutions called the Taskforce on Nature-related Financial Disclosures (TNFD) (Taskforce on Nature-related Financial Disclosures 2024). The final TNFD recommendations were published in September 2024. To promote and realize business and financial organizations that adopt initiatives to accommodate ecological values, citizens’ participation and influence on sustainability policies and businesses is crucial, given the potential role of citizen and stakeholder engagement in sustainability transformation (von Weizsäcker and Wijkman 2017; Carrick et al. 2022).

Danielsen et al. (2024) posit that greater involvement of citizens in GBF would enhance societal engagement in international agreements and enhance quality decision-making based on improved information, leading to effective conservation actions. A recent example of citizen participation concerns the case of marine biodiversity conservation in the European Union (Whyte et al. 2024). Citizen science in the field of biodiversity conservation is also increasing (e.g., Peter et al. 2019; Fraisl et al. 2022; de Groot et al. 2023; Kiruba-Sankar and Barman 2024; Ruck et al. 2024).

However, in terms of citizen participation, there are also concerns regarding the global decline of democracy in the past decade, measured by the varieties of democracy (V-Dem) index, including the participatory component index (Marina et al. 2024). How, then, should we strengthen our capacity for citizen engagement in biodiversity conservation in a less participatory world? To address this problem, it is imperative to consider the interconnectedness between the global and the local on the issues of biodiversity and democracy, both as a problem and a solution. To adapt to the connected issues of ecosystem crises at the global and local levels, citizen participation that connects global and local responses is required. However, such citizen interactions remain limited.

Accordingly, this study explores the emerging opportunity for citizen participation and deliberation, linking the global and the local through cross-cultural citizen dialogue and focusing on the potential capacity development of reflexivity, a significant aspect of deliberation. A deliberative democracy that harnesses inclusive reflection is a prerequisite for sustainability transformation (Dryzek and Pickering 2019; Visseren-Hamakers et al. 2021; Carrick et al. 2022; Shimizu 2022; Nakamura et al. 2023). Reflection includes an individual’s critical self-assessment through dialogue and deliberation, and the system’s self-critical adaptation. Since the 2010s, national and sub-national random sampling-based representative citizen deliberations have emerged in several Organisation for Economic Co-operation and Development (OECD) countries (OECD 2020). The Citizens’ Convention on Climate in France in 2019/2020 and the Climate Assembly UK in 2020 are well known for their national-level citizen deliberations on climate policy (CECS 2019; Climate Assembly UK 2020). The OECD has been promoting the institutionalization of citizen deliberation (OECD 2021), which has extended to transnational and global levels since 2005 (Rask et al. 2019). However, trials of citizen dialogue across national borders remain limited, such as those for European Union-level cases and a recent global-level trial called the Global Assembly on Climate and Ecological Crisis in 2021 (European Parliament 2021; Global Assembly 2023).

Recently, research advancements have been achieved to fill this gap, including the development of a method of reflecting on dialogue and its application to cross-country citizen dialogue on SDGs connecting Finland and Japan (Nakamura et al. 2024). Moreover, research has developed an empirical measurement of attitudinal change for dialogue, boundary of collaboration (an aspect of a sense of global citizenship), and awareness of cultural inheritance that constrains and promotes sustainability transformation, before and after the dialogue. As population-level data on attitude toward dialogue and the boundary of collaboration are available in Taiwan and Japan (Nakamura and Chen 2023), the experimental citizen dialogue across the cultural boundaries of Taiwan and Japan enables comparison between the population data and small-group data of case studies in two specific countries of East Asia.

The current study aims to explore the potential of cross-cultural deliberation by organizing citizen dialogue on biodiversity conservation and citizen role, connecting Taiwan and Japan online. This creates an opportunity for Japanese citizens to learn with their Taiwanese counterparts that share East Asian culture and yet have certain differences, such as Taiwan’s being a more advanced gender equality state in Asia (National Council for Sustainable Development 2022) and citizens’ more active political participation. There have been numerous deliberative democracy practices in Taiwan since the late 1990s, including those commissioned by governmental organizations to pursue democratic legitimacy or to solve political disputes, and those initiated by non-governmental organizations (Fan 2021). According to the V-Dem index 2023, Japan and Taiwan were globally ranked 30th and 31st, respectively, regarding the liberal democracy index; Taiwan ranked 4th for the participatory component, whereas Japan was 69th (Marina et al. 2024). In this study, the mental healthcare approach that originally developed in Finland and Norway is applied to a citizen dialogue. The study thus explores the possibility of increasing an aspect of a sense of global citizenship and awareness of cultural inheritance that may hinder or facilitate transformation for sustainability. The boundary of collaboration, which measures who will collaborate with whom, is a significant characteristic for nurturing a sense of global citizenship under globalization and diversity (Chung and Mitsuyoshi 2017). Yet, less than half of the global population is estimated to have an appropriate order of consciousness or intelligence that would allow them to collaborate with a larger range of people (Kegan 1994; Kegan and Lahey 2009). This project is the first trial of its kind conducted in East Asia.

By connecting Taiwan and Japan, we aim to set the cultural conditions of similarity and difference in East Asia in the current case study. Namely, Taiwanese and Japanese societies share a Confucius culture (Inglehart 2018); however, unlike Japan, Taiwan has advanced political participation and greater gender equality. This cultural setting is expected to offer a modest stimulation—not too large and not too small—between Taiwanese and Japanese participants during dialogue. Moreover, cross-cultural interaction might even stimulate self-reflection among Taiwanese participants, who may be more engaged with societal issues than their Japanese counterparts.

The specific research questions are as follows: What are the impacts of cross-cultural citizen dialogue with the method of reflecting on Taiwanese and Japanese participants’ (a) self-reported recognition of SDGs, (b) self-reported attitude toward dialogue, (c) self-reported boundary of collaboration (an aspect of a sense of global citizenship), and (d) self-reported awareness of cultural inheritance for sustainability?

The remainder of this paper is structured as follows: Sect. “Design of citizen dialogue, attitudinal data, and data analysis” describes the data collection method of citizen dialogue and its analysis, while Sect. “Results” presents the results of citizen dialogue. Section “Discussion” discusses the findings and characteristics of Taiwanese and Japanese participants and their interactions, as well as implications for connected global and local institutions. Finally, Sect. “Conclusions” summarizes the contributions of this study and discusses the perspectives of cross-cultural citizen dialogue research and actions for sustainability transformation.

## Materials and Methods

### Design of citizen dialogue, attitudinal data, and data analysis

#### Design of citizen dialogue

The method of reflecting for the current citizen dialogue was adopted from the practice of mental healthcare, which originated and developed in Norway and Finland (Andersen 1987; Seikkula and Arnkil 2006, 2014; Yahara 2016; Yahara and Andersen 2022). The method of reflecting in this study was adopted from what was used in a previous citizen dialogue tailored for small-group citizen dialogue on policy issues (Nakamura et al. 2024); it was specifically guided by the revealed facts of this method in order to activate participants’ psychological resources (Seikkula and Arnkil 2006, 2014) and secure psychological safety, and ultimately encourage open, constructive criticism with a sense of belonging for all members in an organization (Yahara 2024). The purpose of reflecting was twofold in this study: (1) being heard and responded to and (2) creating a reflecting space. The aim was to nurture the reflecting capacity of participants and allow them to express their voice and respond to it. The method of reflecting, explained below for the case of the current citizen dialogue, is unique despite its resemblance to other facilitation/dialogue methods such as fishbowl. Fishbowl is meant to allow dialogical communication in a large group of people; there is no structured reflection by the people who listen, especially by the people who speak in front of the audience in the circle (The Involve Foundation 2024).

In each round, each group of eight participants was divided into two small-groups (four participants), and the small-groups changed the role of “speaking” and “listening” in turn, as follows: In Session 1, small-group members (“speaking” role) shared their views and ideas on the theme with web camera “on.” During that time, the other small-group members (“listening” role) carefully listened to the dialogue, with web camera “off.” In Session 2, the small-group members who played “listening” role in Session 1, turned on camera, and talked about what they felt and thought, based on what they heard. The small-group members who played “speaking” role in Session 1, listened to the dialogue on their dialogue, with camera “off.” In Session 3, the small-group members who played the “listening” role in Session 2, turned the camera on, and had dialogue on what they felt and thought when they listened to “dialogue about dialogue.” During that period, the other small-group members listened to the dialogue, turning off the camera. All participants were to play the roles of speak-listen-speak and listen-speak-listen through two rounds of group dialogues. Note that, however, two Japanese participants played only the role of speak-listen-speak, while two Taiwanese participants played only the role of listen-speak-listen, for both rounds, due to an accident in Group C in Round 2, which was different from the planned assignment.

Instruction on the style of reflecting was as follows: Talk from the “I”; Connect to the person’s words (“when x said …”), and respond to what has been said; Share tentative offerings, and avoid generalizations (“I wonder if … perhaps … I’m not sure but …”); Brief, 1 to 2 comments per reflector; Slow paced with pauses in between comments; and Reflections can also be phrased as open questions.

Moreover, the organizer requested participants to follow a particular manner of dialogue, as was the case for a series of citizen dialogues on radioactive waste management in post-Fukushima Japan: Do not reject other participants’ remarks; avoid having a single individual control the dialogue process; and let the process unfold without the need to reach an agreement or a conclusion (Nakamura et al. 2021).

For the Taiwan–Japan online citizen dialogue on biodiversity conservation and citizen participation, 24 Taiwanese and 24 Japanese participants, aged 20–69 years for adult Taiwanese and 18–69 years for adult Japanese, were invited from across the countries of residence, following recruitment by a survey company in each country that has a pool of survey respondents (see Table 1). The number of participants was the highest possible for our research resources, but appropriate enough to materialize several small-groups for the current provisional case study. The pools do not guarantee demographic, ideological, and ethnic representation of each society but are not inclined to a specific demographic, ideology, or ethnicity. This is because the only conceivable bias would be an expectation of a reward for participating in the Internet survey, though it is also the case that citizen dialogue participants have a more positive attitude toward dialogue against the population as discussed in Sect. “Self-reported attitude toward dialogue.” The survey companies are general ones, and hence, the individuals of each survey pool are not inclined toward a specific interest such as environmental issues. Apart from gender and age, the participants were selected to represent diverse residential areas of each country, covering both urban and rural areas. The final number of participants was 24 Taiwanese and 22 Japanese individuals (46 in total) because of cancelations of participation on the date of the event. Online citizen dialogues were held for three-and-a-half hours on weekends in September 2024. The time difference between Taiwan and Japan is one hour.

**Table 1:** Gender and age group distribution of Taiwanese and Japanese participants. The number in parentheses show actual participants excluding absentees. *Source* Authors

*Taiwanese*	Age					
	20s	30s	40s	50s	60s	Total
Female	3	3	2	2	2	12
Male	3	3	2	2	2	12
Total	6	6	4	4	4	24

*Japanese*	Age					
	18–29 years	30s	40s	50s	60s	Total
Female	3	3	2	2	2	12
Male	3	3 (2)	2	2	2 (1)	12 (10)
Total	6	6 (5)	4	4	4 (3)	24 (22)

An honorarium of NTD 2 500 (around EUR 70 at the time of the dialogue) was offered to Taiwanese participants and a gift card equivalent to JPY 12 000 (around EUR 75 at the time of the dialogue) to Japanese participants. Participants were paid for participating in an online citizen dialogue and completing the pre- and post-event surveys.

Pre-study materials were sent to participants for preparation, in addition to general guidelines for participation in the event. The main pre-study materials were sections for SDG Goals 14 and 15 (biodiversity conservation) and Goal 16 (peace, justice, and strong institutions, including participatory decision-making) of each country’s latest Voluntary National Review reports on SDGs. Notably, Taiwan is not an official member state of the United Nations; the government has developed and implemented its own SDG program and published Voluntary National Review reports. The Taiwanese reports were written in traditional Chinese and Japanese reports were in Japanese. Additional introductory information on biodiversity issues and nature conservation on the Internet, specific to each country, was also shared. Invitation documents and participation guidelines were written in traditional Chinese for Taiwanese citizens and in Japanese for Japanese citizens.

The program for citizen dialogue is illustrated in Table A1 in supplementary information. There were six groups (A to F, breakout sessions in the Zoom video conference system) comprising eight members. Each group comprised two subgroups of four members. All group members were allocated such that both gender and age diversity were realized in each group in both rounds. Round 1 addressed the theme of biodiversity conservation, while Round 2 explored the citizen role for biodiversity conservation. In Round 1, the subgroups comprised either four Taiwanese or four Japanese, with the two subgroups for each group divided by country. In Round 2, the subgroups were a mixture of citizens from both countries, that is, two Taiwanese and two Japanese individuals. The groups allocated to half of the members were changed for Round 2 so that all participants could experience the role of first speaker in the method of reflecting (details are explained below). The total deliberation time was three hours for two rounds.

For each group, an experienced facilitator was assigned to assist the dialogue and follow the method of reflecting; there were three Taiwanese and three Japanese facilitators. Regarding the language used in the dialogue, a Taiwanese or Japanese interpreter who were able to speak both Chinese and Japanese languages was assigned to each group, and consecutive interpretations (sentence by sentence as well as non-simultaneous interpretations) were made so that citizen participants and facilitators could use their native language. All comments were translated; thus, twice as much time was required to complete the dialogue. See Table A2 in supplementary information for facilitation guidelines. Preparatory online meetings were held between facilitators and researchers to ensure facilitators’ understanding of the method of reflecting, facilitation principles, and purpose of the research.

To guide dialogue among participants, the key facilitation question was presented by the facilitator in group dialogue: “What kind of custom would you want to reconsider in society as a whole, regarding the relationship between humanity and natural environment?” The question was intended to facilitate participants’ reflections on the customs and habits that were conventional in their lives in each country. The question was also presented in the participation guidelines that were sent to participants prior to the dialogue, as part of the preparation to read pre-study materials. During the group dialogue, a simpler version of the facilitation question was also explained by facilitators and shown in Zoom chats in both Chinese and Japanese, so that participants could easily recall the guiding question: “What is the custom that you want to reconsider in daily life, regarding the relationship between humanity and the natural environment? (Round 1)/regarding the relationship among people? (Round 2).” The addition of “in daily life” facilitates participants’ understanding of what was requested and avoids any uncertainty arising from an inability to think abstractly regarding societal issues. The framing to “reconsider” the unconscious custom was maintained.

### Attitudinal data measurement

Pre- and post-event surveys were administered to citizen dialogue participants to measure attitudinal data and their changes before and after dialogue in traditional Chinese for Taiwanese participants and in Japanese for Japanese participants (see Table A3 in supplementary information for the pre-/post-survey questionnaires translated into English). The survey was administered online, using Google Forms. A pre-survey was administered after instruction, and study materials were sent and before the initiation of the citizen dialogue. The participants were asked to answer the prequestionnaire before reading the study materials. A post-survey was administered right after the dialogue event with a three-day allowance. The surveys assessed self-reported knowledge of SDGs, self-reported sense of urgency and significance of biodiversity conservation and participatory decision-making, attitude toward dialogue, self-reported boundary of collaboration defined by trust ratio/score, and self-reported awareness of cultural inheritance toward sustainable development. Information on self-reported overall satisfaction with the event and self-reported evaluation of fair operation were also sought in the post-event survey. Self-reported data are susceptible to the placebo effect and acquiescence bias. Therefore, the change in attitudinal data before and after dialogue should be interpreted with caution. However, a positive self-assessment of the capacity of dialogue and reflection is a prerequisite and is resourceful for actual participation and engagement in dialogue in the collective decision-making process, and it can be observed objectively. A subjective sense of efficacy and confidence is foundational for facilitating dialogical transformation, which can be detected in an objective manner.

Self-reported knowledge of SDGs was measured on a three-point scale: “I do not know,” “I have heard about it,” and “know it.” A sense of urgency and significance of biodiversity conservation (Goals 14 and 15) and participatory decision-making (Target 16.7) were measured on five-point scales as follows: (a) Urgency: “Not urgent,” “Not Urgent, if any,” “Hard to say,” “Urgent, if any,” and “Urgent”; (b) Significance: “Not significant,” “Not significant, if any,” “Hard to say,” “Significant, if any,” and “Significant.”

Self-reported attitude toward dialogue included two components: listening and speaking (Nakamura et al. 2021). Regarding listening, the respondents were asked, “Are you able to listen to others with a view different from your own, regarding social, national, or local issues, without rejecting it, if not accepting it?” Regarding speaking, they were asked, “Are you able to convey your own ideas to others who may have different ideas from yours regarding social, national, or local issues?” Responses were scored on a five-point scale for both questions: “Difficult,” “Somewhat difficult,” “Hard to say,” “Somewhat possible,” and “Possible.”

Self-reported boundary of collaboration was measured using the indicators developed by Nakamura and Chen (2023), who referred to the trust indicator in the World Values Survey (WVS). First, respondents were asked to choose one of the following answers: “Most people can be trusted” and “Need to be very careful,” which was the same as a question measuring general trust in the WVS. Subsequently, to measure the boundary of trust as a basis of collaboration, we asked participants about trust considering 15 social attributes: (A) personal acquaintance, (B) same nationality without acquaintance, (C/D) same/different political orientation, (E/F) same/different economic level, (G/H) same/different professional industry of household income generator, (I/J) same/different religion, (K/L) same/different education level, (M/N) same/different nation/ethnicity, and (O) no attribution. In cases (C)–(O), it was assumed that the nationality of the person was unknown.

Of these 16 attributes in question, there are key reference boundaries of collaboration: person in general (most people), personal acquaintance, unknown person of the same nationality, and unknown person regardless of nationality (no attribution). Personal acquittance is easy for a respondent to visualize specifically, while an unknown person of the same nationality refers to a literally imagined member of the community whom a respondent never meets in reality. An unknown person regardless of nationality indicates the furthest imaginary member of humanity with whom a respondent might have a sense of fellowship to collaborate. The online population surveys in Taiwan and Japan revealed that most respondents chose trust according to closer reference boundaries—that is, acquaintances, unknown person of the same nationality, and unknown person regardless of nationality—and that “person in general” was situated between acquaintances and unknown person of the same nationality (Nakamura and Chen 2023).

An issue is that repetitive survey items risk fatigue or boredom among participants, making the data unreliable. However, this is not a concern, based on existing survey practices, as exemplified by the fact that the WVS uses around 17 organizational attributes regarding institutional trust in a repetitive way, and the interpretation of data was reliable (Haerpfer et al. 2022).

The trust ratio, that is, the number of respondents who chose “trust” divided by the sum of the number of respondents who chose “trust” and the number of respondents who chose “be careful,” was calculated for general trust and each attribute-based trust for all participants. Alternatively, trust score was defined for each respondent for each question, by allocating one (1) for the answer “Most people can be trusted” and zero (0) for “Need to be very careful.” The trust ratio is equivalent to the average trust score for each country for specific attributes of the boundary of trust or collaboration.

Self-reported awareness of cultural inheritance aims to measure the subjective meta-recognition of one’s own cultural tendency that may hinder or promote sustainability transformation. Accordingly, the respondents were asked the following question: Do you agree with the statement below? Please select the most appropriate answer. “I can imagine what cultural difficulties and potentials I inherited as a (Taiwanese/Japanese) person about socially and environmentally sustainable development.” Responses were scored on a five-point scale: “I cannot,” “I cannot, if any,” “Hard to say,” “I can, if any,” and “I can.”

### Data analysis

For each attitudinal variable, changes in distribution were confirmed by the country. Given the small sample size, in addition to statistical tests using *p* value, effect sizes, such as the *ϕ* coefficient and* d* value, were reported as objective, comparable data to observe the effect of participating in citizen dialogue through reflection. A caveat to this study is that the findings cannot be generalized to the population because of the small sample size and use of purposeful sampling. The data obtained from the dialogue participants before participation were compared with the population data surveyed in 2021/2022 for the variables self-reported attitude for dialogue and self-reported boundary of collaboration/trust (Nakamura and Chen 2023).

In addition to the quantitative analysis, content analysis was performed to understand if responses were made in Session 2 to the comments in Session 1 and if reflection on cultural characteristics was made in Session 3 in each round of small-group dialogue (See Sect. “Design of citizen dialogue” to reconfirm what was done in each Session according to the method of reflecting). Twelve rounds were analyzed in this regard. Moreover, qualitative and illustrative reflection by participants was sought through free remarks on the event, which was asked as the final question in the post-event questionnaire survey in order to analyze self-reported attitude for dialogue and self-reported awareness of cultural inheritance for sustainability. Quotations were presented when they match the focus of the analysis. The number of participants who presented such remarks was reported for the analysis of self-reported awareness of cultural inheritance.

## Results

Citizen dialogue showed a high level of satisfaction for both Taiwanese and Japanese participants (see Table 2). Thus, the dialogue met the necessary, if not sufficient, condition of meaningful citizen deliberation.

**Table 2:** Satisfaction and fairness assessment by participants. There is no answer for a Taiwanese participant on fairness assessment. *Sources* Authors

	*Satisfaction*				
	“Not good”	“Not good very much”	“Somewhat good”	“Good”	Total
Taiwanese	0	1	6	17	24
Japanese	0	0	5	17	22
Total	0	1	11	34	46

	*Fairness*					
	“Was not organized in a fair manner”	“Was not organized in a fair manner, if any”	“Hard to say”	“Was organized in a fair manner, if any”	“Was organized in a fair manner”	Total
Taiwanese	0	0	0	4	19	23
Japanese	0	0	0	3	19	22
Total	0	0	0	7	38	45

### Recognition of SDGs: self-reported sense of urgency and significance on biodiversity conservation and citizen participation

**Fig. 1:**
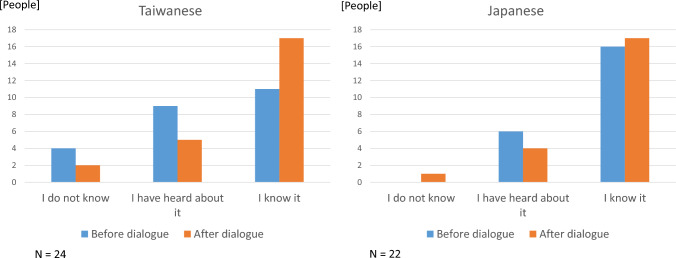
Knowledge of SDGs among participants

**Fig. 2:**
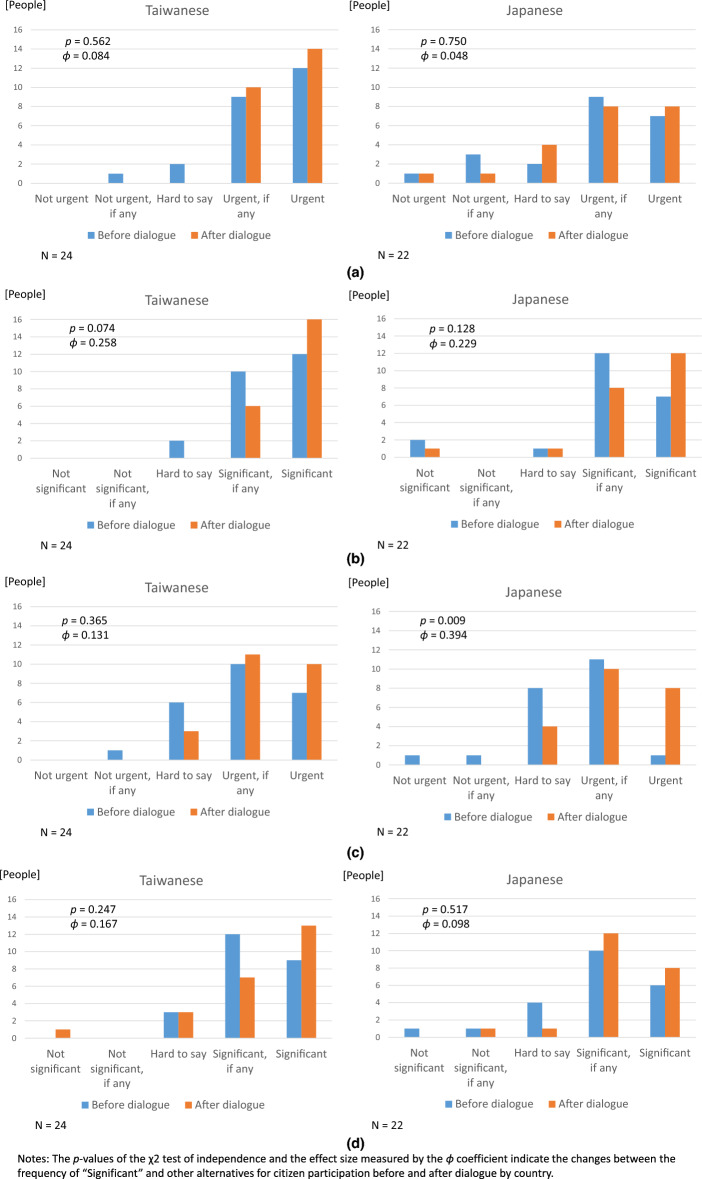
**a** Recognition of biodiversity conservation: urgency, **b** Recognition of biodiversity conservation: Significance, **c** Recognition of citizen participation: urgency, **d** Recognition of citizen participation: Significance. Significance refers to abbrebiation of “a sense of significance” on either biological conservation or citizen participation in such conservation activities. This also holds to “urgency”

As illustrated in Fig. 1, the self-reported recognition of SDGs was higher among Japanese participants (the median is “I have heard about it” for Taiwanese participants, while that is “I know it” for Japanese participants), while it increased for Taiwanese participants after the dialogue. Moreover, as shown in Fig. 2, pre-dialogue self-reported recognition of the sense of urgency and significance of biodiversity conservation was more positive among Taiwanese participants than among Japanese participants (Fig. 2a). The median is between “Urgent, if any” and “Urgent” for Taiwanese, while that is “Urgent, if any” for Japanese, in Fig. 2b. The median is between “Significant, if any” and “Significant” for Taiwanese, while that is “Significant, if any” for Japanese, although the distribution of the self-reported sense of urgency and significance for citizen participation was similar for both Taiwanese and Japanese participants (Fig. 2c). The median is “Urgent, if any” both for Taiwanese and Japanese, in Fig. 2d. The median is “Significant, if any” both for Taiwanese and Japanese.

Moreover, the *p* values of the *χ*^2^ test of independence and the effect size measured by the *ϕ* coefficient indicate the changes between the frequency of “Urgent/Significant” and other alternatives for biodiversity conservation and citizen participation before and after dialogue by country (Fig. 2). The extreme response was used for a comparison with all other responses so that it corresponds to the calculation used in the previous domestic citizen dialogue study in post-Fukushima Japan, where an increase in such responses is meaningful, as the Japanese are said to avoid extreme responses (Nakamura et al. 2021).

Small (0.1–0.3) to medium (0.3–0.5) effects of dialogue on recognition change were suggested in some cases of urgency/significance of biodiversity conservation/citizen participation. In particular, the increased self-reported recognition of significance of biodiversity conservation among Taiwanese participants showed a *p* value less than 0.1 (Fig. 2b). A medium effect was observed for increased self-reported recognition of the urgency of citizen participation among Japanese participants, with the *p* value less than 0.1 (Fig. 2c).

### Self-reported attitude toward dialogue

**Fig. 3:**
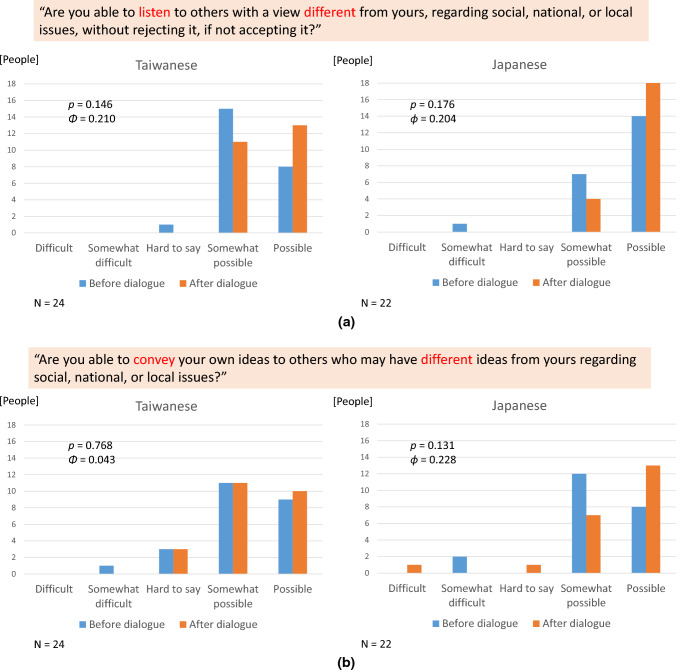
**a** Attitude for dialogue: Listening, **b** Attitude for dialogue: Speaking

Before participating in the citizen dialogue, the self-reported attitude toward listening to different views among Japanese participants was more positive than that among the Taiwanese, while the self-reported attitude toward expressing diverse views was similar for both groups, as demonstrated by the frequency distribution in Fig. 3a. The median is “Somewhat possible” for Taiwanese participants, while that is “Possible” for Japanese participants; (b) the median is “Somewhat possible” both for Taiwanese and Japanese participants. The self-reported attitude toward dialogue among participants in the current citizen dialogue differed from that of the population of each country, as confirmed by a social survey conducted among 800 Japanese citizens in 2021 and 800 Taiwanese citizens in 2022 (Nakamura and Chen 2023). Overall, 95.8% and 83.3% of Taiwanese participants in the current dialogue (before dialogue) showed a positive self-reported attitude toward listening and speaking, respectively, while 65.9% and 65.0% of Taiwanese social survey participants indicated a positive self-reported attitude toward listening and speaking, respectively. Likewise, 95.5% and 60.0% of Japanese participants in the current dialogue (before the dialogue) showed a positive self-reported attitude toward listening and speaking, respectively, while 46.0% and 36.1% of the Japanese social survey participants indicated a positive self-reported attitude toward listening and speaking, respectively. The attitudinal characteristic of more positive self-confidence among participants against the population distribution is understandable and predictable. This difference was also reported in a more rigorous study of Japanese individuals that compared citizen dialogue participants and survey participants who were randomly invited from the citizen register (Nakamura et al. 2021); these participants were completely different from those of the citizen dialogue for the current research.

Regarding the effect of participating in citizen dialogue, the *p* values of the *χ*^2^ test of independence and the effect size measured by the *ϕ* coefficient, indicating the change of dichotomous frequency distribution of “Possible” and other alternatives, before and after dialogue, for listening and speaking capacity self-assessment, by country, are also shown in Fig. 3. Participation in dialogue suggested to have a marginal (0.1–0.3) effect to increase the positive self-reported attitude toward dialogue for both Taiwanese and Japanese participants, except for speaking capacity among Taiwanese participants who already showed strong self-reported capacity before dialogue.

Regarding the Taiwanese participants’ increase in self-reported attitude for dialogue—listening, a Taiwanese participant who chose “possible, if any” after the dialogue reported the reason for positive attitude based on the dialogue experience as follows:

*“All attendees are of high quality and will not be extreme or rude.”* (Translated by the authors) (Male, Taiwanese, 60s)

This would indicate the significance of a direct, bodily experience of quality dialogue for reinforcing or nurturing positive confidence for listening to others who have different views from one’s own.

Regarding the Japanese participants’ increase in self-reported attitude for dialogue—speaking, a Japanese participant’s reason for choosing “possible” after dialogue is indicative of encouragement to learn a potential role of bodily experience through citizen dialogue in sustainability transformation governance:

*“Through today’s dialogue, I bodily felt that we can mutually learn and be aware of new things by means of exchanging views, even if we have different cultural backgrounds. That is why I think I could speak to the people who have different views from my own, believing that I can change my behaviors and attitudes in reference to today’s learning and findings.”* (Translated by the authors) (Male, Japanese, 18–29)

This reflection shows that participation in citizen dialogue could potentially affect some of the participants’ belief in the person’s efficacy and nurture trust in dialogical communication across cultural differences through direct experience.

### Self-reported boundary of collaboration (trust ratio/score)

**Fig. 4:**
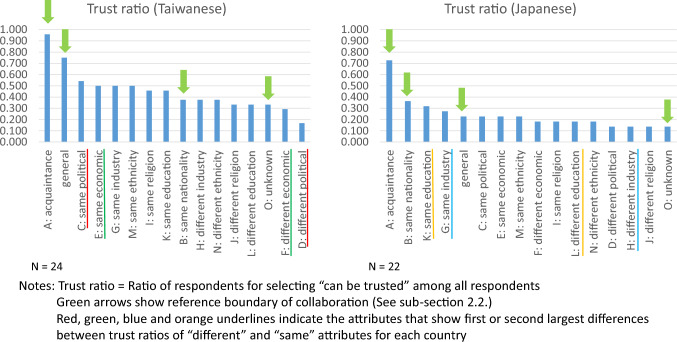
Boundary of collaboration (Trust ratio/score): Pre-dialogue

Figure 4 displays the results of the measurement of pre-dialogue self-reported boundary of collaboration by country, measured by the trust ratio referring to the WVS trust indicators. The trust ratio is calculated as the number of respondents who selected “Can be trusted,” divided by the total number of participants for each country, to measure the trust in people with the same and the different attributes. Taiwanese participants showed higher self-reported trust than Japanese participants in both the general population and other categories of people with specific attributes. Interestingly, the order of trust ratios in the reference boundary of collaboration—that is, “acquaintance,” “person in general,” “unknown person of the same nationality,” and “unknown person regardless of nationality”—was different for both countries. The trust ratio for a person in general was lower than that for an unknown person of the same nationality for Japanese participants. The self-reported boundary of trust or collaboration for a person in general was recognized somewhere between trust for a similar national and a trust for foreigner for Japanese participants, whereas the self-reported boundary of trust for a person in general was perceived between trust for an acquaintance and trust for a same national for Taiwanese participants.

Moreover, difference in six personal attributes, including economic level, led to lower trust ratios than for same personal attributes for all attributions in both countries. However, the characteristics of the gaps between self-reported trust ratios for same and different attributes differed by country. The gaps were largest in political orientation (0.375, difference between C and D, red line in Fig. 4), followed by economic status (0.208, difference between E and F, green line in Fig. 4) among Taiwanese participants, while they were largest in industry of household income generator (0.136, difference between G and H, blue line in Fig. 4) and education level (0.136, difference between K and L, orange line in Fig. 4) among Japanese participants. This indicates that political and economic conditions significantly influence self-reported social trust in Taiwanese society. The gaps generated by the different attributes of self-reported trust were smaller among Japanese participants than their Taiwanese counterparts. The results obtained through current citizen dialogue correspond to the social survey observations of the population in Taiwan and Japan in 2021/2022: Taiwanese people showed higher self-reported trust ratios than their Japanese counterparts. Furthermore, the differences in perceptional attributes led to moderately lower self-reported trust in the professional industry and educational level among Japanese respondents, and political orientation and economic level among Taiwanese respondents (Nakamura and Chen 2023).

Table 3 presents the change in perceived boundary of collaboration before and after dialogue, measured by the average self-reported trust score for each attribute of the person in question, by country. Trust score is defined as one (1) for “Can be trusted” and zero (0) for “Need to be careful.” The difference is calculated as a post-dialogue value subtracted by a pre-dialogue value. The results included the effect size, that is,* d* value or standardized mean difference. The standardized mean difference is calculated as the difference of post-dialogue mean and pre-dialogue mean, divided by the square root of the mean of post-dialogue variance and pre-dialogue variance.

**Table 3:** (a) Boundary of collaboration before and after dialogue for Taiwanese participants, measured by averaged trust score. (b) Boundary of collaboration before and after dialogue for Japanese participants, measured by trust score. Trust score is defined as 1 for “Can be trusted” and 0 for “Need to be careful.” *Source* Authors

Attribute	Pre-dialogue	Post-dialogue	Difference (post–pre)	Effect size (*d* value)
*(a)*				
General	0.750	0.792	0.042	0.099
A: acquaintance	0.958	0.917	− 0.042	− 0.173
B: same nationality	0.375	0.583	0.208	0.426
C: same political	0.542	0.625	0.083	0.170
D: different political	0.167	0.250	0.083	0.206
E: same economic level	0.500	0.542	0.042	0.083
F: different economic level	0.292	0.250	− 0.042	− 0.094
G: same industry	0.500	0.500	0.000	0.000
H: different industry	0.375	0.458	0.083	0.170
I: same religion	0.458	0.667	0.208	0.430
J: different religion	0.333	0.250	− 0.083	− 0.184
K: same education level	0.458	0.542	0.083	0.167
L: different education level	0.333	0.333	0.000	0.000
M: same ethnicity	0.500	0.417	− 0.083	− 0.168
N: different ethnicity	0.375	0.375	0.000	0.000
O: unknown	0.333	0.375	0.042	0.087
*(b)*				
General	0.227	0.318	0.091	0.205
A: acquaintance	0.727	0.909	0.182	0.485
B: same nationality	0.364	0.364	0.000	0.000
C: same political	0.227	0.318	0.091	0.205
D: different political	0.136	0.227	0.091	0.237
E: same economic level	0.227	0.318	0.091	0.205
F: different economic level	0.182	0.182	0.000	0.000
G: same industry	0.273	0.318	0.045	0.100
H: different industry	0.136	0.273	0.136	0.343
I: same religion	0.182	0.318	0.136	0.319
J: different religion	0.136	0.227	0.091	0.237
K: same education level	0.318	0.318	0.000	0.000
L: different education level	0.182	0.227	0.045	0.113
M: same ethnicity	0.227	0.318	0.091	0.205
N: different ethnicity	0.182	0.318	0.136	0.319
O: unknown	0.136	0.273	0.136	0.343

Among Taiwanese participants, small (0.2–0.4) effect to increase self-reported trust were indicated for attribute of “different political orientation” which showed largest trust score gap against “same political orientation” before dialogue. Among Japanese participants, small (0.2–0.4) effects to increase self-reported trust were observed for four attributes, including an attribution that had a large gap between different and same attributes, namely “different industry.” Moreover, the self-reported trust of an unknown person, regardless of nationality, which was lowest before dialogue, also increased.

### Self-reported awareness of cultural inheritance for sustainability with participants’ voices of reflection

**Fig. 5:**
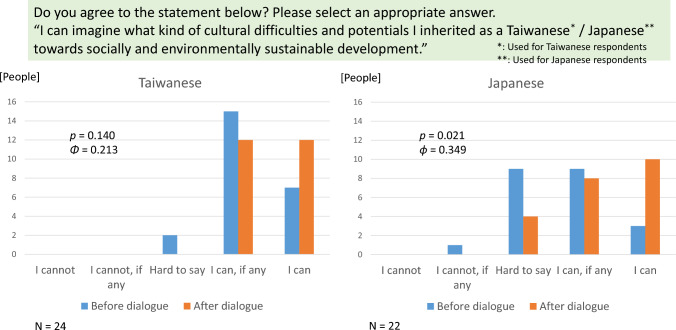
Awareness of cultural inheritance

Before participating in dialogue, Taiwanese participants showed a more positive self-assessment than the Japanese for awareness of cultural inheritance as difficulties and potentials for sustainability transformation, as shown in Fig. 5. (The mode is “I can, if any” for Taiwanese participants, while that is between “Hard to say” and “I can, if any” for Japanese participants, though the median is “I can, if any” for both Taiwanese and Japanese participants.)

The *p* values of the *χ*^2^ test of independence, and the effect size (*538*), to summarize the dichotomous distributions of self-reported awareness of cultural inheritance (“able to think” and other alternatives) before and after dialogue, by country, are also reported in Fig. 5. Clearly, participation in citizen dialogue through reflection suggested to have a small-to-medium (0.2–0.4) effect of increasing self-reported awareness of cultural inheritance for both Taiwanese and Japanese participants. In particular, the effect for Japanese participants showed a *p* value less than 0.05.

Participants’ free comments in the post-dialogue survey also depict an increased self-reported awareness of the cultural inheritance of sustainability transformation. Examples of such reflections are as follows:

*“I was able to recognize my personal and my country’s problems that are otherwise not easily acknowledged in daily life. I believe I can make the best use of this learning in my daily life.”* (Translated by the authors) (Female, Japanese, 18–29)

*“The event allowed participants to exchange opinions, fostering a greater understanding that biodiversity and environmental protection transcend national borders.”* (Translated by the authors) (Male, Taiwanese, 50s)

These two remarks resonate with each other, highlighting the reflective nature of cross-cultural dialogue in daily life and transcending the nature of contemporary human activities and biodiversity degradation.

As revealed by the larger effect size compared with Taiwanese participants, the increase in self-reported awareness of cultural inheritance for sustainability was greater among Japanese participants. This tendency is also supported by post-dialogue remarks from Japanese participants, as follows:

*“In my group, I felt Taiwanese people had serious interest in and commitment to the issues, and I myself need to think more about environmental problems.”* (Translated by the authors) (Male, Japanese, 40s)

*“To be honest, I was not interested in the topic at the beginning. However, I got many ideas while I was speaking, and many others agreed with the ideas that I shared with them. The conversation with the people from a different country was refreshing and interesting.” *(Translated by the authors) (Male, Japanese, 30s)

These remarks indicate the susceptibility and preparedness of several Japanese participants to accept perceptional change in what they can do, which they are unaware of during their daily lives. Moreover, in a free answer for reasoning of overall satisfaction of the dialogue event, 18 of 22 Japanese participants presented their views, touching on Taiwan, Taiwanese topics, or notion of different countries/cultures. This might also indicate an appropriateness of cross-cultural dialogue reflecting on one’s own culture as stimulation and exercise, even if it takes as short a period as around three hours online.

### Response to utterances: state of reflecting among participants

The purpose of applying the method of reflecting to citizen dialogue was to realize the opportunity for participants to be heard and responded to by other participants. To assess the consequences, we analyzed the records of group dialogue and the state of responses. Table 4 summarizes the topics spoken about in Session 1 that were responded to in Session 2 for each round. This confirms that there were relevant responses in Session 2 that were directly linked to the utterances made in Session 1 in all rounds of group dialogue, regardless of the themes of biodiversity conservation and citizen participation. It is also noteworthy that this reflective response occurred across the diverse topics discussed in the different groups.

**Table 4:** Topics in Session 1 responded to in Session 2 in each round of dialogue. *Source* Authors

Group	Round 1 (Biodiversity conservation)	Round 2 (Citizen role in biodiversity conservation)
A	•Straw management at cafés for the environment •Plastic use and marine life •Wind power and clean energy •Vegetarianism and insect eating	•Effects of entertainment and movies to engage people in conservation •Further governmental information dissemination •Economic incentive provision for scaling up citizen participation
B	•Japanese consumers’ actions, such as plastic bag refusal and material recycling, to protect biodiversity	•Increasing youth engagement through social networking services is needed to protect ocean environment
C	•Importance of a sense of ownership for protecting biodiversity •Individual opinion dissemination through the Internet •Firefly restoration in a Taiwanese forest park	•Citizen participation in forest conservation •Authentic interest in biodiversity conservation and SDGs among citizens •Exotic pets and invasive alien species
D	•Segregating waste and recycling at households •Mass production, consumption, and wastage •Impact of individual practices such as refusing plastic bags •Impact of clothing industry	•Big picture thinking of Taiwanese participants to understand Japanese behaviors •Potential positive use of peer pressure to promote conservation
E	•Different roles of individuals and governments •Waste and plastic management for ocean environment •Forest management and urban greening •Enterprise role and funding for conservation activities	•Citizen role in promoting governmental action; for example, Kaohsiung City in Taiwan •Citizen and individual action starting from clear local needs
F	•Commitment of private companies to biodiversity conservation; ocean environment •Seeking both environmental conservation and economic sustainability •Connected people have power to change •Dialogue and discussion could change ideas •Connection between waste and marine environment •Necessary roles of government, companies and citizens	•Importance of indigenous people and culture to protect the environment •Economic incentive for bringing your own cup •Effectiveness of school education for nature conservation across generations •Small, yet important power of citizens •Importance of value change rather than economic incentives

Realizing that “being heard” and “being responded to” are part of the dialogue forms the basis to facilitate and prompt participants’ authentic reflection of daily practices and beliefs that they were unaware of in daily lives. Without a sense of trust, safety, and care in dialogue, a meaningful internal reflection process cannot be initiated (Yahara 2024). Considering the constraints in understanding and limited time allocation because of interpretation, challenges in concentration in online communication, and mechanical/technical problems, the minimum condition of trust-building for reflective dialogue might have been met, since there were ample, relevant responses for both rounds for all six groups.

## Discussion

### Self-reported attitude toward dialogue

As illustrated in Figs. 1 and 2, the self-reported sense of urgency and significance of biodiversity conservation and citizen participation was stronger among Taiwanese participants before dialogue than their Japanese counterparts, although the average self-reported knowledge level regarding SDGs was higher among Japanese participants. The potential medium-level effect of dialogue participation on the increase in the self-reported sense of urgency of ensuring “responsive, inclusive, participatory and representative decision-making at all levels” among Japanese participants post-dialogue, indicated by *p* value and effect size (*φ *coefficient) shown in Fig. 2c, might be explained by a hypothetical impact of small-group-level interaction with Taiwanese individuals for Japanese participants. Indeed, Taiwanese people recently showed active participation at the collective level (Fan 2021; Marina et al. 2024). The Public Policy Online Participation Platform (join.gov.tw), established by Taiwan’s National Development Council in 2015, enables citizens to propose creative ideas and engage in discussions on public policies, either individually or as part of a group. Incorporating participatory budgeting increases public expression and mutual understanding of public issues while fostering communication between citizens and the government. This mechanism may contribute to increasing public awareness of issues related to SDGs and encouraging citizens to listen to and express their views in Taiwan.

Moreover, participating in citizen dialogue across cultural borders might have had a small effect on making self-reported attitudes toward dialogue with different views more positive, both for listening and speaking, except for speaking among Taiwanese individuals, who already displayed a more positive self-reported attitude before the dialogue. Therefore, the effect of strengthening a culture of dialogue was suggested for Taiwan–Japan dialogue, as was the case for another cross-cultural dialogue on gender equality and biodiversity conservation between Finland and Japan (Nakamura et al. 2024) and domestic dialogue on radioactive waste management within Japan (Nakamura et al. 2021). The apparently non-significant effect of dialogue on self-reported speaking attitude among Taiwanese participants could be interpreted as a saturation phenomenon; that is, the self-reported attitude that was already positive pre-dialogue does not significantly increase post-dialogue. This phenomenon was also observed in Japanese participants’ self-reported attitudes toward listening in a Finland–Japan citizen dialogue (Nakamura et al. 2024). All these data show that participation in citizen dialogue might contribute to the capacity development of group members who have a weaker self-reported attitude toward dialogue than the counterpart group members. In the current study, Taiwanese participants might have strengthened their self-reported capacity to listen on average, while Japanese participants might have reinforced their self-reported capacity to speak on average, beyond the fluctuation by chance.

### Self-reported boundary of collaboration (trust ratio/score)

As elucidated in Sect. “Self-reported boundary of collaboration (trust ratio/score),” Japanese participants indicated an extended self-reported boundary of collaboration or a higher self-reported trust ratio/score toward a hypothetical person with four different attributes from the respondent, on average. Their Taiwanese counterparts also displayed a small extension of self-reported collaboration boundaries for one different attribute that showed the largest gap of trust ratio/score against the same attribute. As the latter showed higher average self-reported trust ratios than their Japanese counterparts by approximately 0.2 (see Fig. 4 and Table 3 for overall tendency), there may have been a saturation phenomenon, as is the case for self-reported attitudes toward dialogue. A saturation phenomenon was also observed in the Finland–Japan citizen dialogue, where Finnish participants showed higher self-reported trust ratios than their Japanese counterparts pre-dialogue, and did not indicate a significant increase in their self-reported trust ratios post-dialogue. Therefore, from these two explorative case studies, we can infer that cross-cultural citizen dialogue participation could weakly extend the self-reported boundary of collaboration of participants from the cultural group that has a collectively narrower self-reported boundary of collaboration than its counterpart, as measured by the self-reported trust ratio/score. As confirmed above, Taiwanese individuals have a wider self-reported boundary of collaboration than Japanese individuals. Therefore, cross-cultural interaction between them would contribute to cultivating a subjective aspect of a sense of global citizenship among the Japanese toward biodiversity conservation and sustainability transformation in general.

Furthermore, compared with the effect of cross-cultural citizen dialogue on the self-reported awareness of cultural inheritance for sustainability, the effect on the self-reported boundary of collaboration might have been weaker. From the perspective of transcending cultural (and national) boundaries, practicing and training awareness regarding one’s own culture seems more direct than broadening the perceptional boundaries of collaboration. To achieve SDGs, a subjective aspect of a sense of global citizenship is a clear target for citizenship education in the contemporary world. However, more direct experiences of cross-cultural collaboration than dialogue are required to elicit a stronger effect on transforming embedded beliefs regarding the self-reported boundaries of collaboration.

### Self-reported awareness of cultural inheritance for sustainability

As exemplified in Sect. “Self-reported awareness of cultural inheritance for sustainability with participants’ voices of reflection” and Table 4, participation in cross-cultural dialogue might have resulted in a small-to-medium-level effect on increasing self-reporting awareness of cultural inheritance, which may hinder and facilitate sustainability transformation. This hypothetical effect of cultivating self-reporting awareness of one’s own culture, which would be required for systemic, perceptional, and structural transition from an unsustainable socio-ecological to a sustainable state (von Weizsäcker and Wijkman 2017), aligns with previous findings from the Finland–Japan online citizen dialogue on SDGs (Nakamura et al. 2024). The hypothetical effect for Finnish and Japanese participants in the Finland–Japan citizen dialogue was larger than that for Taiwanese and Japanese participants in the Taiwan–Japan citizen dialogue in the present study, presumably because of weaker self-assessment of awareness of cultural inheritance pre-dialogue for the participants in the Finland–Japan citizen dialogue. Based on these two case studies, cross-cultural citizen dialogue with a method of reflecting might enhance self-reported awareness of cultural inheritance for sustainability transformation for participants from both cultural groups regardless of their pre-dialogue level of awareness. In addition, a cross-cultural dialogue with a method of reflecting would bring about a stronger self-reported attitude toward dialogue for the group who showed weaker pre-dialogue attitude compared with their counterparts. The potential positive effect on the capacity of reflection at the group level regardless of the pre-dialogue level would be a promising finding to nurture not only a culture of dialogue with individuals with diverse views but also a culture of reflection toward sustainability transformation, for it would be desirable that all individuals recognize their own cultural constraints and resources for necessary structural change. This practice might identify and cultivate individual- and small-group-level capacity for conscious observation and decisions on what to change and what to inherit from the perspective of sustainable development.

Since the preliminary test, the hypothetical effect measured by the effect size shown in Fig. 5 applies to both the Japanese and Taiwanese participants. The cross-cultural dialogue might cultivate the space needed for improvement in terms of collective “self-awareness”—for the people in both groups—although the impact might be larger among people with weaker self-reported awareness (Japanese participants, in this case study). This mechanism differs from a unilateral, asymmetrical transfer of awareness capacity from a stronger group to a weaker group. The cross-cultural dialogue could function as a time and place of mutual learning for all participants.

### Policy implications for international institutions of cross-cultural citizen dialogue

GBF explicitly stipulates a theory of change as a vehicle for implementing the framework: “The Kunming-Montreal global biodiversity framework is built around a theory of change which recognizes that urgent policy action is required globally, regionally and nationally to achieve sustainable development so that the drivers of undesirable change that have exacerbated biodiversity loss will be reduced and/or reversed to allow for the recovery of all ecosystems and to achieve the Convention’s Vision of Living in Harmony with Nature by 2050” (Convention on Biological Diversity 2022). A core of the theory of change is a causal relationship statement with goals, followed by a pathway that usually includes vision, mission, and program (Funnell and Rogers 2011). Necessary policy actions that connect the global and local toward halting biodiversity loss and recovering ecosystems follow the vision of awareness and transformation for sustainability. Such policy innovations include randomly invited citizen assemblies as institutionalized deliberation platforms for democratic transnational and global governance (Bohman 2010; Dryzek et al. 2011). To directly cultivate such a capacity for reflection as a foundation for sustainability transformation, it would be worthwhile to consider institutionalizing cross-cultural online citizen dialogue as a part of international institutions for sustainable development, including biodiversity conservation. Such a dialogue process can be connected to and followed by a formal collective decision-making process, as demonstrated and advocated at the sub-national, national, and supranational levels (Bouricius 2013; Van Reybrouck 2013; OECD 2020, 2021). It is time to initiate and explore an effective institution at the global level that directly connects citizens at the local level. Local, here, means both sub-national and national. We can recall the proposal of substantial interstate collaboration relying on our sense of global nation-ship (or homo sapience-ship), that is, one global nation with many states, which materializes effective, de facto world government through collaborative governance, as exemplified by a sense of global connection and one-ness during the COVID-19 pandemic (Damluji 2019).

Cultivating cultural awareness and the ability to transform (un-)sustainability can be institutionally embedded in the process of dialogue and deliberation in formal collective decision-making. A dialogical reflecting process can be designed upstream of a series of processes, followed by downstream of policy option formulation and final decision-making (Escobar 2011; Bouricius 2013; Van Reybrouck 2013). Such an international institutional setting contributes to nurturing an open dialogue culture not only at the organizational and national levels, but also at the global level, connecting small-group interaction and national and global institutions for sustainability awareness and reflexive transformation (Nakamura et al. 2023). As the potential effectiveness of self-reported awareness capacity building has been hinted at in previous and current case studies, such exploration can be applied not only for citizen interaction but also for experts and decision-makers, emphasizing the potential hindering effect of power gradient and imbalance among participants on dialogue and reflection (Nakamura et al 2023).

The possible realization of the vision above regarding biodiversity conservation includes two-track global interactions that connect formal and informal channels, wherein citizens have opportunities to engage in dialogue and deliberation across cultural borders. One is an online global citizen dialogue on biodiversity conservation, focusing on monitoring and financing issues that are outstanding challenges from COP16, as an input for global and national decision-making for implementing the Convention of Biological Diversity. This is an attempt to follow the United Nations’ design process of SDGs or the voice of Future We Want, which is more inclusive and participatory than ever before in terms of the number of events and space provided to hear the voices of as many stakeholders as possible. The other is an online global citizen dialogue organized by networks of civil society organizations or sub-national governments aiming to directly foster and develop an exchange of citizen voices, regardless of the official positions of the states worldwide. Such examples include a project of on-site citizen deliberation called WWViews on biodiversity conservation in various countries, simultaneously conducted by a network of civil society organizations (Rask and Worthington 2015). This is in line with the current initiative to establish a permanent Global Citizen’s Assembly for People and Planet to address humanity’s greatest challenges, as an extension of Global Assembly on the Climate and Ecological Crisis in 2021 (Iswe Foundation 2024). These two tracks of dialogue processes can be either dependent and closely connected, or independent. Both cases are meaningful as deliberative systems and polyphonic dialogical space developments connecting the global and local levels (Seikkula and Arnkil 2006; Dryzek 2000, 2010).

### Limitations and further research

This was an explorative case study with a limited sample size, and participants were not invited through comprehensive random sample sorting. Even for the case of Taiwan–Japan citizen dialogue, further attempts with larger sample size during repeated small-group dialogue operation by various organizations are desirable. The findings of this research should be treated as hypotheses to be tested rather than as hypotheses already tested by the data we obtained. Given the selection biases for the participants of this citizen dialogue event, a potential change in various attitudinal variables is also a preliminary possibility that could be applied only to the people with such bias. However, the candidates running for the election are also biased toward participation, even as political representatives for society. This also holds even for random sampling for citizen’s assembly or mini-publics: There is a selection bias for those who accept such an invitation by sortition against those who do not accept such an offer to be a representative of a community. Nonetheless, mini-publics are much more representative than other forms of political representation. Moreover, from the viewpoint of individual and societal capacity development, as well as diversified, distributed willingness to participate in collective decision-making process, such a bias is not necessarily undesirable, but inevitable, and, more importantly, a desirable target of capacity development for society. When more people have such a bias, more people are prone to participate, and society would have a higher participatory democracy score in V-Dem. Thus, arguments of selection bias, and associated issues of data subjectivity, or being non-objective, should be cautiously and explicitly described and discussed along with the purpose of empirical, observation-oriented research and practice, that is, sustainability transformation governance in the current research context.

The long-term impact of cross-cultural dialogue on self-reported attitudinal variables of participants is another research focus in settings of more popular, regular implementation and utilization of citizen dialogue in collective decision-making. Indirect effects on political efficacy, trust in institutions, and participatory attitudes of non-participants in citizen dialogue in regions where citizen dialogue is institutionalized are also to be studied. Moreover, cross-cultural citizen dialogue other than that between Taiwan and Japan in East Asia, such as that between South Korea and Japan or tripartite citizen dialogue among Taiwan, South Korea, and Japan, could be investigated. The third party’s voice may have a triangulation, facilitation role to overcome potential difficulties arising from bilateral historical and cultural relationships and to cultivate reflective space during the dialogue.

Therefore, two research directions are recommended. One is to increase the number of cross-cultural citizen dialogues on SDGs and other societally significant issues, with the method of reflecting and with an empirical self-reported attitudinal variable measurement before and after the dialogue. This leads to foundational database development and maintenance to support the realization of deliberative democracy and participatory environmental governance research and practice at the global, national, and sub-national levels, as exemplified by examples such as the OECD database on mini-publics, V-Dem, WVS, and International Social Survey Programme. In doing so, future research should answer whether there is support for cross-cultural citizen dialogue that is worth the investment of resources and difficulty of interpretation. Controlled experiments comparing cross-cultural and enclave deliberation in line with the existing literature (e.g., Grönlund et al. 2015) are expected. Moreover, the research could be extended to post-dialogue reflection by the project team members—citizen participants and/or researchers and other staffs, referring to another study on the use of reflective practice for sustainability transition (Boyle et al. 2021)—or reflective action research on post-disaster volcanic disaster management (Nakamura et al. 2019).

The second is to design research and experimental execution of cross-cultural citizen dialogue in actual global, national, and sub-national institutional settings to bridge the gap between small-scale case studies and population-level decision-making processes.

## Conclusions

This study empirically explored the possibility of nurturing and cultivating self-reported reflexivity capacity at the small-group level through cross-cultural online citizen dialogue on biodiversity conservation and citizen role with a method of reflecting, connecting Taiwan and Japan. Reflexivity capacity both at the individual and small-group level and at the collective, population level is required for sustainability transformation. This study found that cross-cultural dialogue might have improved self-reported awareness of cultural inheritance as a hindering and promoting factor for sustainability transformation in one’s own cultural community, and expanded the perceived boundary of collaboration measured by trust score/ratio. Other findings include the positive shift in self-reported attitude toward dialogue with a person holding different views and of urgency/significance assessment of sustainability transformation issues, such as biodiversity conservation (ecological, economic problems, and current state) and citizen participation (solution to be materialized by a problem owner). The mean treatment effect of cross-cultural dialogue with the method of reflecting on self-reported cultural inheritance awareness was suggested for both Taiwanese and Japanese participants. By contrast, the effect on self-reported attitude toward dialogue, self-reported boundary of collaboration, and the self-reported urgency/significance evaluation of sustainability issues were suggested mainly for the cultural group that showed weaker self-assessment against counterpart cultural groups before the dialogue. Overall, these findings present the necessity of further rigorous, systematic investigation of the effectiveness of cross-cultural online citizen dialogue with a method of reflecting on more participatory, reflexive sustainability governance, in the context of East Asian culture. The initiative of cross-cultural citizen dialogue demonstrates the potential that we could “think locally, act globally” and “think globally, act locally” simultaneously, when citizen dialogue is embedded in local and global action planning and execution.

The topics of this study’s cross-cultural citizen dialogue were biodiversity conservation and citizen role in realizing conservation. Our daily, structural, cultural beliefs and actions may hinder or promote the required transformation. As such, redefining the human being as a part of the whole ecosystem, and regenerating the relationship among people, is a necessary challenge toward sustainability transformation. Accordingly, embedding cross-cultural citizen dialogue in formal, informal, and non-formal collective decision-making institutions globally is an urgent issue given the rapidly destabilizing and decaying biological and social environment in recent decades (von Weizsäcker and Wijkman 2017; United Nations 2023; Marina et al. 2024). This line of citizen dialogue might restore democracy and nurture positive political discourse and beliefs. Comprehensive, inclusive research on cultivating a capacity for reflection both at the small-group and society-as-a-whole level would be desirable. The current research demonstrates a subtle, yet clear step toward the development of sustainability transformation governance research.

## Supplementary Information

Below is the link to the electronic supplementary material.

Supplementary file1 (PDF 398 KB)

## Data Availability

There are no supplementary materials. Raw data used for statistical analysis are available upon request.
