# Supplementary material for: Online citizen dialogue on biodiversity conservation and citizen participation: A cross-cultural deliberation between Taiwan and Japan
Source: Ambio. 2025 Jul 9;54(12):2170–89. doi: 10.1007/s13280-025-02209-6 (PMC12569313; doi:10.1007/s13280-025-02209-6)
Supplement: Supplementary file 1 — Supplementary file1 (PDF 398 KB) [file 13280_2025_2209_MOESM1_ESM.pdf]

Ambio

Supplementary Information
This supplementary information has not been peer reviewed.

Title: **Online citizen dialogue on biodiversity conservation and citizen participation: A cross-cultural deliberation between Taiwan and Japan**

Table A1. Program of Taiwan-Japan online citizen dialogue on biodiversity conservation and citizen participation

Taiwanese time	Japanese time	Activities
13:20 – 13:30	14:20 – 14:30	Reception
13:30 – 13:40	14:30 – 14:40	Opening, Introduction
13:40 – 15:10	14:40 – 16:10	Group dialogue on biodiversity conservation (Round 1)
15:10 – 15:20	16:10 – 16:20	Break
15:20 – 16:50	16:20 – 17:50	Group dialogue on citizen role for biodiversity conservation (Round 2)
16:50 – 17:00	17:50 – 18:00	Closing

Sources: Authors

Date: Sunday, September 22, 2024

Time difference between Taiwan and Japan: 1 hour

Table A2. Facilitation guideline

Principles

- Facilitator (one person per group) develops and maintains a field of dialogue (listening each other even if they do not agree and expressing themselves honestly) with minimal intervention so that citizen participants can have smooth dialogue in their group dialogues.
- Facilitator guides the method of Reflecting (shown below), keeps time, and develops the field (breathing slowly; being aware of what they are speaking; and observing other participants' responses on their own talk) where participants would obtain observations and findings, through dialogue about dialogue.

Introduction and topic setting

- At the beginning of the group dialogue, self-introduction is asked (Check-in). Since half of participants change the groups in Round 2, self-introduction is held in Round 2, too. Let participants introduce names and their motivation of participation.
- When needed, the following explanation of theme settings and reasons (stated in Participation Guideline) are explained by facilitator: "Regarding biodiversity, the Kunming-Montreal Global Biodiversity Framework was adopted and the United Nations Biodiversity Conference in December in 2022. Vision 2050 and new global targets to achieve by 2030, including effective conservation and management of at least 30 per cent of the world's land, coastal areas and oceans were developed."
- Let them talk based on their experiences and views, not necessarily based on expert knowledge.
- They can talk either based on pre-study materials distributed before the dialogue, or on any other information sources and personal experiences.
- Participants are encouraged to talk from a global, national and individual perspective for biodiversity.
- Guiding question to think: "What kind of custom would you want to reconsider in society as a whole, regarding relationship between humanity and natural environment?"
- Facilitator will put the following questions, both in Chinese and in Japanese, in Zoom Chat at the beginning of the group dialogue session 1 for Round 1 (biodiversity conservation) and Round 2 (roles of citizens for biodiversity conservation), so that participants reconfirm the question during the group dialogue:
- Round 1: "人間と自然環境との関係について、どのような習慣を社会全体で問い直してみたいだろうか," "作為一個社會，我們希望重新考慮人類與自然環境的關係什麼樣的慣習？"
- Round 2: "人間どうしのつながりについて、どのような習慣を社会全体で問い直してみたいだろうか," "作為一個社會，我們希望重新考慮人人類之間的聯繫什麼樣的慣習？"
- At the same time, let them touch upon concerns and different views and ideas on current / proposed actions for biodiversity conservation, if any.
- If no participants start to talk, let them review the pre-study materials, and facilitate talking. Open questions such as "How do you want to use the time of

group dialogue?" or "How can we start the dialogue?" might be used.

Way of dialogue: Reflecting

- Each group (eight participants) is divided into two small groups (four participants), and the small groups change the role of "speaking" and "listening" in turn, as follows:
    1) Small group members ("speaking" role) share their views and ideas, and have a dialogue, on the theme (what then felt and thought on pre-study materials and others, significance, necessity of treatment, and others), with web camera "on." During that time, the other small group members ("listening" role) actively listen the dialogue, with web camera "off." (30 minutes)
    2) The small group members who played "listening" role in 1), turn on camera, and talk about what they felt and thought, "based on what they actually heard." In that response, let them express their views, not decisively, using such words as "perhaps" "maybe." Moreover, encourage them to speak slowly. The small group members who played "speaking" role in 1), listen to the dialogue on their dialogue, with camera "off." (22 minutes)
    3) The small group members who played "listening" role in 2), turn on camera, and have dialogue on what they felt and think when they listened to "dialogue about dialogue." During the period, the other small group members listen to the dialogue, turning off the camera. (22 minutes)
- These three sessions, in total around 75 minutes, constitute one round. After one round completes, another round of reflecting is held, changing the roles of "speaking" and "listening." Half of participants will change their groups, or Zoom breakout rooms, when they have group dialogue for biodiversity. The small groups are made of solely either Taiwanese or Japanese citizens when they talk about "biodiversity conservation" that is social theme, while the small groups are comprised of both of Taiwanese and Japanese citizens when the theme is "role of citizens for biodiversity conservation."

Manner of dialogue

- Follow the manner of dialogue "do not deny; do not dominate the dialogue; and do not (need to) reach conclusion."
- Encourage to actively listen to what the speaker says. It is allowed to make questions for clarification and understanding.
- When there are remarks to negate the other participants' views, encourage the speaker to share the background ideas, experiences and values so that such difference can be received (but not agreed) by other participants, and to deepen dialogue.
- Do not intervene rather long utterance when the dialogue is felt being deepen. If assessed needed, intervene, saying such as "It is becoming a bit long." "May we listen to other persons (or Mr/Ms ….)?"

Response to questions to facilitator

- Do not say facilitators' views and opinions (if requested to do so)
- When receiving questions on factual things, do not make concrete answers. Ask if any participants know the answers.

Source: Authors

Table A3(a). Pre-dialogue questionnaire

1. On SDGs

1-1. Do you know the United Nations Sustainable Development Goals (SDGs)? Please select an appropriate answer.
(1) I do not know. (2) I have heard about it. (3) I know it.

1-2. Please provide your personal views on urgency and significance to strive on the following SDGs, compared to the other social problems. Please select an appropriate answer.

1-2-1. SDGs 14 & 15: Life below water & life on land
(a) Urgency
(1) Not urgent (2) Not Urgent, if any (3) Hard to say (4) Urgent, if any (5) Urgent

(b) Significance
(1) Not significant (2) Not significant, if any (3) Hard to say (4) Significant, if any (5) Significant

1-2-2. SDG 16: Peace, justice and strong institutions, in particular target 16-7 "Ensure responsive, inclusive, participatory and representative decision-making at all levels"
(a) Urgency
(1) Not urgent (2) Not urgent, if any (3) Hard to say (4) Urgent, if any (5) Urgent

(b) Significance
(1) Not significant (2) Not significant, if any (3) Hard to say (4) Significant, if any (5) Significant

1-3. Do you agree to the statement below? Please select an appropriate answer.
"I can imagine what kind of cultural difficulties and potentials I inherited as a Taiwanese / Japanese* towards socially and environmentally sustainable development."

(1) I cannot. (2) I cannot, if any. (3) Hard to say. (4) I can, if any. (5) I can.

* "Taiwanese" is used for Taiwanese participants, and "Japanese" is used for Japanese participants.

2. On dialogue

2-1. Are you able to listen to others with a view different from yours, regarding social, national, or local issues, without rejecting it, if not accepting it? Please select an appropriate answer. Please also describe the reasons.

(1) Difficult (2) Somewhat difficult (3) Hard to say
(4) Somewhat possible (5) Possible

Reasons

2-2. Are you able to convey your own ideas to others who may have different ideas from yours regarding social, national, or local issues? Please select an appropriate answer. Please also describe the reasons.

(1) Difficult (2) Somewhat difficult (3) Hard to say
(4) Somewhat possible (5) Possible

Reasons

3. On trust

3-1 Generally speaking, would you say that most people can be trusted or that you need to be very careful in dealing with people? Please select an appropriate answer.

(1) Most people can be trusted.
(2) Need to be very careful.

3-2. We would like to ask you how much you trust people from various groups. Could you answer for each whether you trust people from this group? Please select an appropriate answer.

Person in question	(1) Can be trusted	(2) Need to be careful
(A) Personal acquaintance		
(B) **Unknown**, **same** nationality		
(C) **Unknown**, **same** political orientation, regardless of nationality		
(D) **Unknown**, **different** political orientation, regardless of nationality		
(E) **Unknown**, **same** economic level, regardless of nationality		
(F) **Unknown**, **different** economic level, regardless of nationality		
(G) **Unknown**, **same** industry, regardless of nationality		
(H) **Unknown**, **different** industry, regardless of nationality		
(I) **Unknown**, **same** religion, regardless of nationality		
(J) **Unknown**, **different** religion, regardless of nationality		
(K) **Unknown**, **same** education level, regardless of nationality		
(L) **Unknown**, **different** education level, regardless of nationality		
(M) **Unknown**, **same** ethnicity, regardless of nationality		
(N) **Unknown**, **different** ethnicity, regardless of nationality		

(O) Unknown, regardless of nationality		

4. About yourself

4-1 Are you interested in climate change? Please select most appropriate answer.
(1) No. (2) No, if any. (3) Hard so say.
(4) Yes, if any. (5) Yes.

4-2 Please specify your gender.
(1) male (2) female (3) others

4-3 Please specify your age.
(1) 20−29 (2) 30−39 (3) 40−49 (4) 50−59 (5) 60−69 [For Taiwanese]

(1) 18−29 (2) 30−39 (3) 40−49 (4) 50−59 (5) 60−69 [For Japanese]

4-4 Please provide your participation ID that was sent to you beforehand so that your answers before and after participating in citizen dialogue can be compared.

Participant ID:____________________

Thank you very much for your cooperation to the questionnaire.

Table A3(b). Post-dialogue questionnaire

1. On SDGs

1-1. Do you know the United Nations Sustainable Development Goals (SDGs)? Please select an appropriate answer.
(1) I do not know. (2) I have heard about it. (3) I know it.

1-2. Please provide your personal views on urgency and significance to strive on the following SDGs, compared to the other social problems. Please select an appropriate answer.

1-2-1. SDGs 14 & 15: Life below water & life on land
(a) Urgency
(1) Not urgent (2) Not Urgent, if any (3) Hard to say (4) Urgent, if any (5) Urgent

(b) Significance
(1) Not significant (2) Not significant, if any (3) Hard to say (4) Significant, if any
(5) Significant

1-2-2. SDG 16: Peace, justice and strong institutions, in particular target 16-7 "Ensure responsive, inclusive, participatory and representative decision-making at all levels"
(a) Urgency
(1) Not urgent (2) Not urgent, if any (3) Hard to say (4) Urgent, if any (5) Urgent

(b) Significance
(1) Not significant (2) Not significant, if any (3) Hard to say (4) Significant, if any
(5) Significant

1-3. Do you agree to the statement below? Please select an appropriate answer.
"I can imagine what kind of cultural difficulties and potentials I inherited as a Taiwanese / Japanese* towards socially and environmentally sustainable development."

(1) I cannot. (2) I cannot, if any. (3) Hard to say. (4) I can, if any. (5) I can.

* "Taiwanese" is used for Taiwanese participants, and "Japanese" is used for Japanese participants.

2. On dialogue

2-1. Are you able to listen to others with a view different from yours, regarding social, national, or local issues, without rejecting it, if not accepting it? Please select an appropriate answer. Please also describe the reasons.

(1) Difficult (2) Somewhat difficult (3) Hard to say
(4) Somewhat possible (5) Possible

Reasons

2-2. Are you able to convey your own ideas to others who may have different ideas from yours regarding social, national, or local issues? Please select an appropriate answer. Please also describe the reasons.

(1) Difficult (2) Somewhat difficult (3) Hard to say
(4) Somewhat possible (5) Possible

Reasons

3. On trust

3-1 Generally speaking, would you say that most people can be trusted or that you need to be very careful in dealing with people? Please select an appropriate answer.

(1) Most people can be trusted.
(2) Need to be very careful.

3-2. We would like to ask you how much you trust people from various groups. Could you answer for each whether you trust people from this group? Please select an appropriate answer.

Person in question	(1) Can be trusted	(2) Need to be careful
(A) Personal acquaintance		
(B) **Unknown**, **same** nationality		
(C) **Unknown**, **same** political orientation, regardless of nationality		
(D) **Unknown**, **different** political orientation, regardless of nationality		
(E) **Unknown**, **same** economic level, regardless of nationality		
(F) **Unknown**, **different** economic level, regardless of nationality		
(G) **Unknown**, **same** industry, regardless of nationality		
(H) **Unknown**, **different** industry, regardless of nationality		
(I) **Unknown**, **same** religion, regardless of nationality		
(J) **Unknown**, **different** religion, regardless of nationality		
(K) **Unknown**, **same** education level, regardless of nationality		
(L) **Unknown**, **different** education level, regardless of nationality		
(M) **Unknown**, **same** ethnicity, regardless of nationality		
(N) **Unknown**, **different** ethnicity, regardless of nationality		

(O) Unknown, regardless of nationality		

4. On citizen dialogue

4-1. How do you feel your experience of participating in the citizen dialogue this time? Please select an appropriate answer. Please also provide the reasons.

(1) Not good (2) Not good very much (3) Somewhat good (4) Good

Reasons

4-2. Do you think the dialogue was organized in fair and neutral manner? Please select an appropriate answer.
(1) Was not organized in fair manner. (2) Was not organized in fair manner, if any.
(3) Hard to say.
(4) Was organized in fair manner, if any. (5) Was organized in fair manner.

4-3 Did you feel it was difficult to speak your views since it was minority's view among the group members? Please select an appropriate answer.
(1) Frequently (2) Sometimes (3) Almost none (4) None

4-4. Did you feel the dialogue was dominated by the person who has strong views? Please select an appropriate answer.
(1) Frequently (2) Sometimes (3) Almost none (4) None

4-5. Do you think the number of dialogue participants was appropriate? Please select an appropriate answer.
(1) The more, the better. (2) Appropriate (3) The less, the better.
(4) I do not know.

4-6. Were you able to have a dialogue with respect to the others who may have different positions than yours? Please select an appropriate answer.
(1) Was able to do. (2) Was able to do, mostly. (3) Was not able to do very much.
(4) Was not able to do.

4-7-1. Were you able to listen to the others properly? Please select an appropriate answer.
(1) Was able to do. (2) Was able to do, mostly. (3) Was not able to do very much.
(4) Was not able to do.

4-7-2. Did you speak sufficiently? Please select an appropriate answer.
(1) Was able to do. (2) Was able to do, mostly. (3) Was not able to do very much.
(4) Was not able to do.

4-7-3. Did you think a lot? Please select an appropriate answer.
(1) I did. (2) I did, mostly. (3) I did not very much. (4) I did not.

4-7-4. Were we concentrated in dialogue? Please select an appropriate answer.
(1) We did.   (2) We did, mostly.   (3) We did not very much.   (4) We did not.

4-7-5. Did we explore the theme well? Did we have a good discussion? Please select an appropriate answer.
(1) We did.   (2) We did, mostly.   (3) We did not very much.   (4) We did not.

4-7-6. Did you learn new things? Did you get new questions and ideas? Please select an appropriate answer.
(1) I did.   (2) I did, mostly.   (3) I did not very much.   (4) I did not.

4-7-7. Was the dialogue interesting and fun? Please select an appropriate answer.
(1) It was interesting.   (2) It was mostly interesting.
(3) It was not interesting very much.   (4) It was not interesting.

5. About yourself

5-1. Please provide your participant ID that was sent to you beforehand so that your answers before and after participating in citizen dialogue can be compared.

Participant ID:____________________

5-2. Please feel free to provide reflections on this citizen dialogue event.

Thank you very much for your cooperation to the questionnaire.
